# Droplet digital polymerase chain reaction (ddPCR) assays integrated with an internal control for quantification of bovine, porcine, chicken and turkey species in food and feed

**DOI:** 10.1371/journal.pone.0182872

**Published:** 2017-08-10

**Authors:** Hanan R. Shehata, Jiping Li, Shu Chen, Helen Redda, Shumei Cheng, Nicole Tabujara, Honghong Li, Keith Warriner, Robert Hanner

**Affiliations:** 1 Department of Integrative Biology, University of Guelph, Guelph, Ontario, Canada; 2 Biodiversity Institute of Ontario, University of Guelph, Guelph, Ontario, Canada; 3 Microbiology Department, Mansoura University, Mansoura, Egypt; 4 Laboratory Services Division, University of Guelph, Guelph, Ontario, Canada; 5 Department of Food Science, University of Guelph, Guelph, Ontario, Canada; Istituto di Biologia e Biotecnologia Agraria Consiglio Nazionale delle Ricerche, ITALY

## Abstract

Food adulteration and feed contamination are significant issues in the food/feed industry, especially for meat products. Reliable techniques are needed to monitor these issues. Droplet Digital PCR (ddPCR) assays were developed and evaluated for detection and quantification of bovine, porcine, chicken and turkey DNA in food and feed samples. The ddPCR methods were designed based on mitochondrial DNA sequences and integrated with an artificial recombinant plasmid DNA to control variabilities in PCR procedures. The specificity of the ddPCR assays was confirmed by testing both target species and additional 18 non-target species. Linear regression established a detection range between 79 and 33200 copies of the target molecule from 0.26 to 176 pg of fresh animal tissue DNA with a coefficient of determination (R^2^) of 0.997–0.999. The quantification ranges of the methods for testing fortified heat-processed food and feed samples were 0.05–3.0% (wt/wt) for the bovine and turkey targets, and 0.01–1.0% (wt/wt) for pork and chicken targets. Our methods demonstrated acceptable repeatability and reproducibility for the analytical process for food and feed samples. Internal validation of the PCR process was monitored using a control chart for 74 consecutive ddPCR runs for quantifying bovine DNA. A matrix effect was observed while establishing calibration curves with the matrix type under testing, and the inclusion of an internal control in DNA extraction provides a useful means to overcome this effect. DNA degradation caused by heating, sonication or Taq I restriction enzyme digestion was found to reduce ddPCR readings by as much as 4.5 fold. The results illustrated the applicability of the methods to quantify meat species in food and feed samples without the need for a standard curve, and to potentially support enforcement activities for food authentication and feed control. Standard reference materials matching typical manufacturing processes are needed for future validation of ddPCR assays for absolute quantification of meat species.

## Introduction

Food adulteration or mislabeling remains a key challenge to the food industry [1]. The majority of food adulteration is economically motivated whereby low cost ingredients are substituted for high value products [1]. Beyond the economic impact of food fraud, issues of food safety, food contamination and religious restrictions are also relevant [1–3]. Amongst the commodity groups, meat represents one of the most common foods implicated in food fraud [4]. For example, 2013 witnessed the largest single case of food fraud when horse meat was substituted for beef in meat products distributed in the United Kingdom and Ireland [5]. Other studies reported a high incidence of meat substitution, including a 57% substitution rate in processed meat products, a 54% substitution rate in chicken sausages from Italian markets [6, 7], and a 78% substitution rate in beef and poultry products in Malaysia [2]. Examples of substitution included beef and pork in chicken products, and pork in beef products [2]. With respect to animal feed, the inclusion of prohibited species, such as bovine tissues, represents a feed safety issue due to the potential transfer of prions. Feed control regulations have been enforced in many countries since the ruminant feed ban in 1997 in order to minimize the risks of spreading of bovine spongiform encephalopathy (BSE) [8]. The feed industry produces millions of tons of feed, as well as similar amounts of ruminant animal protein-based materials, for non-ruminant feed or other uses each year. Risks of cross-contamination exist on farms and throughout the various stages of production and shipment.

In order to protect the food and feed industry and consumers, regulatory bodies need to monitor food authenticity and feed contamination by ruminant materials. Reliable, rapid and accurate methods are needed for use in food authentication and in feed control [9]. Several techniques have been used for meat species identification, including chromatographic, immunological and electrophoretic methods, but these methods are limited due to their low accuracy, low sensitivity and time consuming processes [9–12]. Furthermore, some of these techniques are unreliable for use with processed or cooked meat [9, 11]. Conventional PCR has been widely used as a qualitative measure of a target DNA whereas quantitative real-time PCR (qPCR) can also quantify copies of the target sequence [2, 8, 9, 12–22]. A recently developed PCR technology is digital PCR (dPCR) which provides more accurate quantification of target DNA [23]. In dPCR, the PCR reaction mixture is partitioned into tens of thousands of droplets with each droplet harboring an independent PCR reaction. The target DNA copy number is determined based on the number of droplets positive for amplification of the target DNA [4, 24, 25]. The dPCR approach provides absolute DNA quantification, eliminates the need for a standard curve as in qPCR, and improves accuracy for quantifying target DNA especially at low concentrations or in a high background of foreign DNA [4, 26]. Many dPCR methods have been developed for quantifying clinical diagnostic targets [27, 28] and for viral, bacterial and parasitic pathogens [29–32]. Recently, dPCR methods have also been reported for quantification of genetically modified organisms (GMO) in food and feed samples [33]. Additionally some ddPCR assays were developed for pork and chicken in meat products using non- mitochondrial genes [4, 24].

The objective of this study is to develop, optimize and validate common droplet digital PCR (ddPCR) assays integrated with an internal control for detection and quantification of bovine, porcine, chicken and turkey species in meat products and animal feed samples. Mitochondrial DNA was selected as the PCR target due to its high copy numbers present in cells, facilitating detection of trace quantities in food and feed matrices, especially when these commodities may be subject to extensive processing. To our knowledge, this is the first comprehensive report to describe ddPCR assays integrated with an internal control to quantify bovine, chicken, porcine and turkey DNA in food and feed samples.

## Materials and methods

### Materials

All fresh raw animal tissue samples, raw milk, blood and plant samples were obtained either from a local grocery store or from Animal Health Laboratory Services, University of Guelph, Ontario. Dry feed samples were obtained from local feed processing plants in Ontario. Pure fresh beef, pork, chicken and turkey muscle tissues and poultry and pork meal feed samples were used as reference materials. The species identities of all materials were confirmed by DNA barcoding based on the standard CO1 gene [34]. The feed samples were confirmed negative for the detection target by PCR for use as matrices for spiking experiments. Fortified samples were prepared from single species materials at known concentrations of the targets and were used as positive controls. A rabbit or fish muscle tissue sample, an unfortified feed sample matrix, reagent blank and PCR water were used as negative controls. An artificial DNA fragment (134 bp) was cloned into a plasmid using pcr4 TOPO cloning kit (Applied Biosystems, Foster City, CA) and used as an internal control (Table 1) [35]. The controls were included in each ddPCR run. Additional non-target species samples used for specificity testing included common birds, mammals, fish and plant species (duck, sheep, goat, dog, horse, mouse, rat, rabbit, Pollock fish, salmon, sole fish, rice, corn, wheat and soybean, Table 2).

**Table 1:** Primers and probes used for ddPCR assays in this study.

Target (Gene)	Primer name	Sequence (5’-3’)^a^	Length (bp)	Accession number	Position on reference sequence	Reference
**Chicken** **(cytb)**	Cytb-F	TCTGGGCTTAACTCTCATACTCACC	106	L08376	690–714	[36]
	Cytb-R	GGTTACTAGTGGGTTTGCTGGG			774–795	[36]
	Cytb-Probe	CATTCCTAACACTAGCCCTA			716–735	[36]
**Porcine** **(ATPase)**	F7773	CTCAATGGTATGCCACAACTAG	313	AF034253	8950–8971	[37]
	R8064	CATTGTTGGATCGAGATTGTGC			9241–9262	[37]
	Porcine-Probe	ATCTCAAACTACTCATACCCAGCAAGCCCA			9040–9069	This study
**Turkey** **(12S rRNA)**	12S-FW	CCACCTAGAGGAGCCTGTTCTGTAAT	122	KP171707	90–115	[38]^b^
	12S-RV2	TTGAGCTCACTATTGATCTTTCATTTT			185–211	[38]^b^
	12S-Probe	TCCACCCAACCACCTCTTGCCAACAC			129–154	This study
**Bovine** **(ATP synthase)**	F8108	CCATATACTCTCCTTGGTGAC	270	KC153975	8108–8128	[37]
	R8357	GTAGGCTTGGGAATAGTACGA			8357–8377	[37]
	Bovine-Probe	TAGACACGTCAACATGACTGACAATGATC			8139–8167	This study
**Internal control** **(Artificial)**	IC-F	AAGACATTGTGGATGCAGATGAGTA	134		1–25	This study
	IC-R	TAGGCAAGTGCATCCTCCTC			115–134	
	IC-Probe	CTTGTCCCTCCTGTTGGTACTAGAGA			27–52	
	IC-Fragment	AAGACATTGTGGATGCAGATGAGTATCTTG TCCCTCCTGTTGGTACTAGAGAGGGGGAAA GGGCGAATTCTGCAAGATGAAAGGGCCCTA CAGATTCGCAGAATTCGCGTGATGGAGGAG GATGCACTTGCCTA				

^a^Probes were labeled with 6-FAM or Cal Fluor Orange (for IC) at 5’ end.

^b^Minor modifications were made to the published primers.

**Table 2:** Samples used and results for evaluating the specificity of ddPCR assays.

	ddPCR target					ddPCR target			
Samples^a^^,^^b^	Porcine	Chicken	Turkey	Bovine	Samples^a^^,^^b^	Porcine	Chicken	Turkey	Bovine
**Target samples**					**Target samples**				
Bovine blood	-	-	-	+	Turkey burgers	-	-	+	-
Bovine heart	-	-	-	+	Turkey hot dogs	-	-	+	-
Beef meat (cooked) -2	-	-	-	+	Turkey kidney	-	-	+	-
Beef meat (raw) -2	-	-	-	+	Turkey liver	-	-	+	-
Bovine milk	-	-	-	+	Turkey meat (cooked)	-	-	+	-
Beef salami	-	-	-	+	Turkey meat balls	-	-	+	-
Beef wiener	-	-	-	+	Turkey meat tissue -2	-	-	+	-
Bovine liver	-	-	-	+	Turkey sausage	-	-	+	-
Chicken breast (cooked)	-	+	-	**-**	**Non-target animal samples**				
Chicken breast fillers	-	+	-	-	Dog meat tissue	-	-	-	-
Chicken heart	-	+	-	-	Duck meat tissue	-	-	-	-
Chicken hot dogs	-	+	-	-	Goat meat tissue	-	-	-	-
Chicken liver	-	+	-	-	Horse meat tissue	-	-	-	-
Chicken meat (raw) -3	-	+	-	-	Mouse meat tissue	-	-	-	-
Chicken wieners	-	+	-	-	Rabbit meat tissue	-	-	-	-
Chicken-pork bologna	+	+	-	-	Rat meat tissue	-	-	-	-
Pork ham	+	-	-	-	Sheep meat tissue	-	-	-	-
Porcine kidney	+	-	-	**-**	**Non-target fish samples**				
Porcine liver	+	-	-	-	Pollock fish	-	-	-	-
Porcine liver sausage	+	-	-	-	Salmon	-	-	-	-
Pork meat (cooked)	+	-	-	-	Sole fish	-	-	-	-
Pork meat (raw) -2	+	-	-	**-**	**Non-target plant samples**				
Porcine spleen	+	-	-	-	Corn	-	-	-	-
Pork summer sausage	+	-	-	-	Rice	-	-	-	-
Pork-beef salami	+	-	-	-	Wheat	-	-	-	-
Pork-chicken bologna	+	+	-	-	Soybean	-	-	-	-
Turkey bacon	-	-	+	-					

^a^The number (n) after an animal or food sample indicates that multiple (n) samples from different individual animals were tested.

^b^Species ID was confirmed for representative target species and all non-target animal and fish species by DNA barcoding.

### Sample preparation

Fresh meat samples were first trimmed of skin and excess fat, and deboned (if applicable), and then cut into ~1.0–1.5 cm^3^ pieces. The meat pieces were placed in a Cuisinart grinder and homogenized for 3–5 minutes. The homogenized samples were then used for DNA extraction. Cooked meat was prepared by autoclaving the meat pieces for 15 minutes at 121°C, and 17.5 psig pressure. The meat samples were allowed to cool, homogenized using a Cuisinart grinder, and then air dried for 72 hours at room temperature (approximately 22°C) or dried in an oven at 70°C for 24 hours to a moisture level of 4–5%. After drying, the samples were ground using a mortar and pestle and passed through a sieve (mesh no. 100) to obtain fine powder. The moisture content was measured using an air-oven method following AOAC 983.18 [39, 40] for sample preparation and AOAC 950.46 Part B [41], air drying, section (a) for sample testing.

Fortified food or feed samples were prepared in a mortar by mixing/homogenizing the appropriate mass of the heat-processed and dried pure meat species powder (500 mg dry wt) with a pure meat, food or feed sample of a different species (4500 mg or 9500 mg dry wt) to obtain 10% and 5% of target species samples initially. Fortified samples at lower concentrations were prepared by mixing 1000–2000 mg (dry wt) of a homogenate at a higher concentration with 1000–4000 mg (dry wt) pure meat, food or feed powder samples accordingly. The samples were portioned into appropriate numbers of 100 mg sub-samples in 1.5 mL micro-centrifuge tubes for testing, or frozen in a -80°C freezer for testing at a later date.

### DNA extraction

Total genomic DNA was extracted from a sub-sample (100 mg) of a homogenized representative food or feed specimen using DNeasy Blood and Tissue^®^ Kit (Qiagen, Mississauga, ON, Canada) following the manufacturer’s protocol. DNA concentrations and quality, including A260 and A280, were measured using a NanoDrop ND-2000 UV Vis ND-2000 Spectrophotometer (Thermo Fisher Scientific, Ottawa, ON, Canada) and a Qubit Fluorometer and Qubit dsDNA BR Assay Kit (Thermo Fisher Scientific). Extracted DNA samples were diluted to a concentration of 10 ng/μL prior to use, or frozen in a -20°C freezer for use at a later date.

### Primers and probes

Primers and probes (Table 1) were selected or designed based on the mitochondrial DNA sequences of the bovine, porcine, chicken and turkey genomes, and 5’-nuclease assay chemistry [36–38]. The primers and probes were designed using the Primer Express Software v3.0 (Applied Biosystems) and synthesized using an ABI 3900 HT synthesizer (Applied Biosystems) at the Laboratory Services, University of Guelph, Guelph, ON. Probes were 5’-labeled with 6-carboxyfluorescein (6-FAM) or Cal Fluor Orange (for the internal control) as the reporter and BHQ-1 as the 3’-labelled quencher. Target genes, primer names, primer sequences, positions on reference sequences, accession numbers and amplicon length are provided in Table 1.

### Droplet digital polymerase chain reaction (ddPCR)

To optimize internal control use in ddPCR assays, 10-fold serial dilutions of the internal control plasmid DNA, corresponding to 0.06–60,000 fg/μL, were prepared, mixed with Bovine DNA (2.0 ng/μL) in a 1:4 ratio, and tested using the ddPCR in duplicates. A higher level of the IC (>4500 copies/PCR) caused competitive amplification with the target DNA (S1 Table), while a low concentration IC was found not stable upon repeated freeze/thaw cycles. The optimal level of the IC was determined to be approximately 1700 ±20% copies per PCR reaction, which provided reliable ddPCR outputs for use as a quantitative measure for each of the ddPCR reactions. The repeatability of the IC ddPCR was evaluated in 16 replicates (S1 Fig). Furthermore, the specificity of the internal control primers and probes was confirmed as described in the ddPCR assay evaluation section below.

The ddPCR reaction conditions were optimized using varied amounts of target DNA in the presence of other non-target species. Optimization experiments included optimizing annealing temperatures (53–63°C) using a Gradient T100 Thermal Cycler (Bio-Rad, Mississauga, ON, Canada), and varied concentrations of primers (400–1000 nM), and probes (200 nM—500 nM). The optimized PCR reaction mixture (25 μL/reaction) contained 1x ddPCR Supermix for Probe (Bio-Rad), 96 nM each of the primers and 64 nM probe for the animal target, 40 nM each of the primers and 32 nM probe for the internal control, 1700 copies of internal control plasmid DNA and 40–50 ng of template DNA. From each PCR reaction mixture, 20 μL were mixed with 70 μL of Droplet Generation oil for Probes (Bio-Rad) in a DG8 Cartridge (Bio-Rad). The cartridge was covered with a DG8 gasket for ddPCR and loaded into the QX200 Droplet Generator (Bio-Rad) to generate PCR droplets. From each droplet mix, 20 μL were then transferred to a 96-well PCR plate (Bio-Rad). The plate was sealed with a foil heat seal using PX1^™^ PCR plate Sealer (Bio-Rad). PCR thermal cycling was conducted using a GeneAmp^™^ PCR System 9700 (Applied Biosystems), following optimized cycling conditions: an initial incubation at 95°C for 10 min, 48 cycles of 20 s at 95°C and 40 s at 59–60°C, followed by a final incubation at 98°C for 10 min and holding at 10°C until reading time. The amplification signals were read using the QX200^™^ Droplet Reader and analyzed using its associated QuantaSoft software (Bio-Rad) and recorded as copies/μL with confidence intervals of 95%. The results from 13,000 or more droplets were accepted and converted into % by weight or by DNA mass for reporting. A ddPCR result was considered acceptable only if the IC gave the expected output with a ≤20% variation.

### ddPCR assay evaluation

To evaluate the ddPCR assays for testing food and feed samples, the assays were tested for their specificity, quantification range, repeatability, reproducibility, matrix effect, robustness and effect of DNA degradation. All experiments were conducted in duplicate unless otherwise indicated. The specificity of the ddPCR assays, including the IC ddPCR, was confirmed *in silico* and *in vitro* by testing both target and non-target species samples (Table 2). The target species samples included raw and processed products from bovine, porcine, chicken and turkey origins. The non-target samples included common animal, fish and plant species (duck, sheep, goat, dog, horse, mouse, rat, rabbit, Pollock fish, salmon, sole fish, rice, corn, wheat and soybean). To evaluate the linearity of the ddPCR assays, different concentrations of DNA were used in multiple PCR reactions. The amounts of DNA used per reaction were three fold dilutions between 1.3 and 320.0 pg for beef, 1.3 and106.8 pg for chicken, 2.2 and 532.0 pg for pork, and, 0.26 and 64 pg for turkey. To test for the effect of tissue type on ddPCR, fresh bovine and porcine skeletal muscle, liver, heart or kidney, were tested in parallel. The quantification range and limit were determined using a series of bovine, porcine, chicken or turkey-fortified food and feed samples with concentrations of 0.005%, 0.01%, 0.05%, 0.1%, 0.5%, 1%, 3%, 5% and 10.00% (wt/wt) and tested in 4 replicates. The repeatability of ddPCR (consistency of results between DNA extraction and between PCR reactions) was determined using the fortified food and feed samples at 5 different target concentrations within the quantification range in 4 replicates. Similarly, intra-lab validation was conducted using three independent trials performed on different days by different operators with a total of 12 data points per concentration per fortified sample for 5 different target concentrations per sample type. The matrix effect was evaluated using cooked animal tissue spiked into different types of food and feed within their respective quantification ranges. For example, chicken meat was spiked into pork summer sausage, beef hot dog or beef and pork salami.

To test the robustness of the assays, the methods were evaluated under different conditions that can be variable under laboratory practices, including DNA storage (fresh versus frozen for 3 weeks), prolonged PCR reagent shelf life (fresh versus old PCR master mix with a few days of shelf life prior to expiry date) and different PCR machines (two systems with different ages but same brand). The samples used were bovine, porcine or chicken-fortified feed or food at 5 or more concentration levels within the quantitative ranges of the assays (S2 Table). The effect of DNA degradation on ddPCR readings was tested using raw and cooked beef DNA after 3 different degradation treatments, Taq I digestion, sonication (42 KHZ) for 2 min and sonication (42 KHZ) for 10 min using a Branson 1510 sonicator (Fisher Scientific, Ottawa, ON). The ddPCR output was compared to the readings from DNA without degradation treatment.

Statistical analyses were performed using chi-square test or paired sample t-test with SAS 9.4 software program to reveal quantification variation significance against different matrices and experimental parameters. GraphPad Prism 6 was used to determine linearity and draw graphs.

## Results

### Specificity

Specificity of the primers and probes was tested first *in silico* using the Nucleotide Basic Local Alignment Search Tool (BLAST, http://blast.ncbi.nlm.nih.gov/Blast.cgi). The specificity of the ddPCR assays, including the internal control ddPCR assay, was further tested experimentally using DNA from target and non-target species samples. The ddPCR assays yielded positive results when target DNA was tested using the corresponding primers/probe while non-target DNA was negative, indicating no cross amplification. The results confirmed the specificity of the ddPCR assays for selective detection of bovine, porcine, chicken and turkey species (Table 2) as well as the internal control within its specificity range (Table 1).

### Linearity and limit/range of quantification

**Fig 1:**
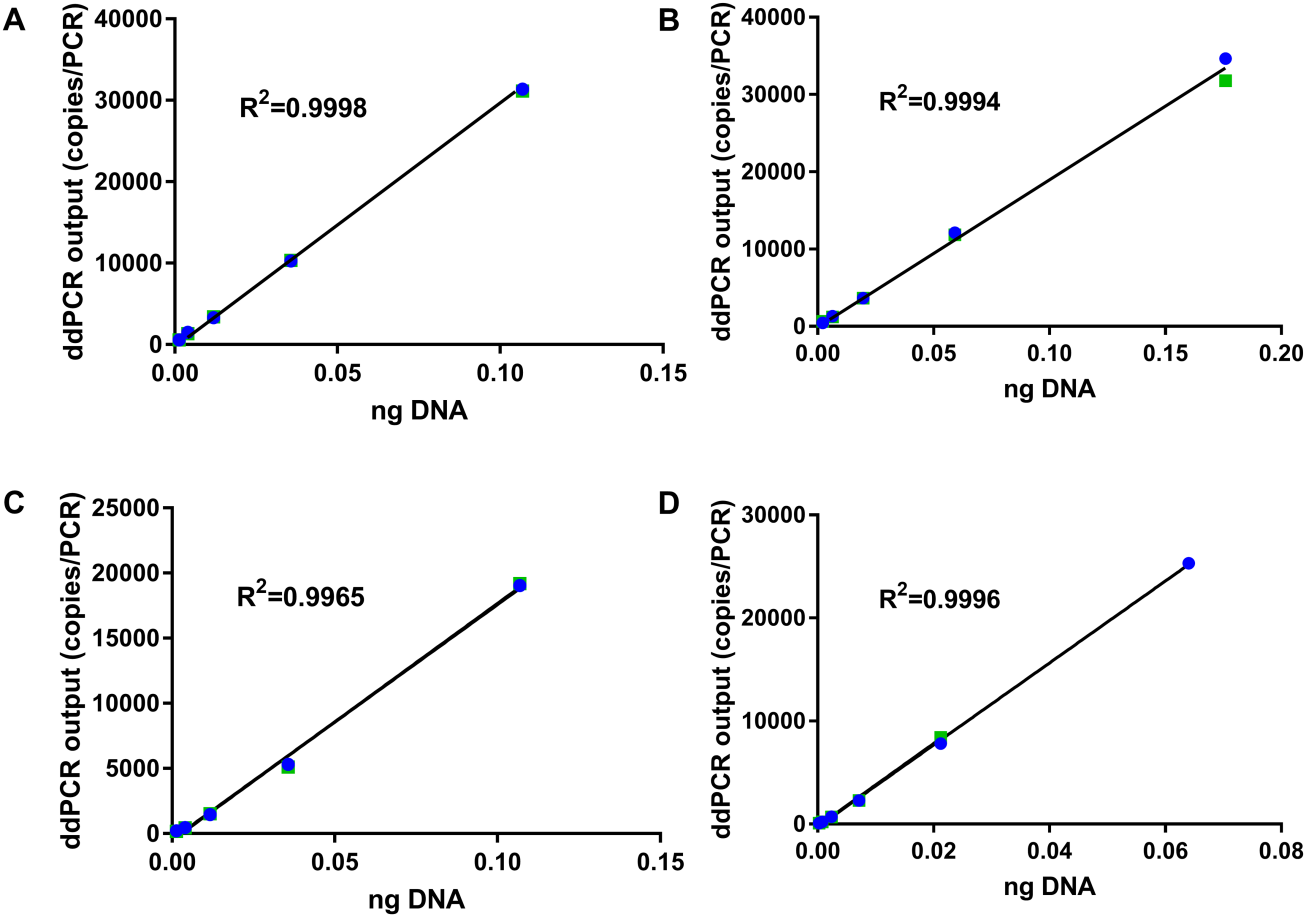
Linear regression between ddPCR output (copies/PCR) and DNA amount (ng). DNA was extracted from fresh raw (A) beef, (B) pork, (C) chicken, and (D) turkey. Shown are results from two replicates in green and blue colors.

The linearity of the optimized ddPCR assays was determined using purified DNA from fresh meat tissue at concentrations of 0.26–532 pg/PCR. The linear regression was established within the concentrations of 1.3–106.8 pg/PCR for beef and chicken, 2.2–176.0 pg/PCR for pork, and 0.26–64 pg/PCR for turkey. These ranges of concentrations were equivalent to 79–33200 copies/PCR, with coefficients of determination (R^2^) ranging from 0.997–0.999 (p-value < 0.0001) (Fig 1).

**Fig 2:**
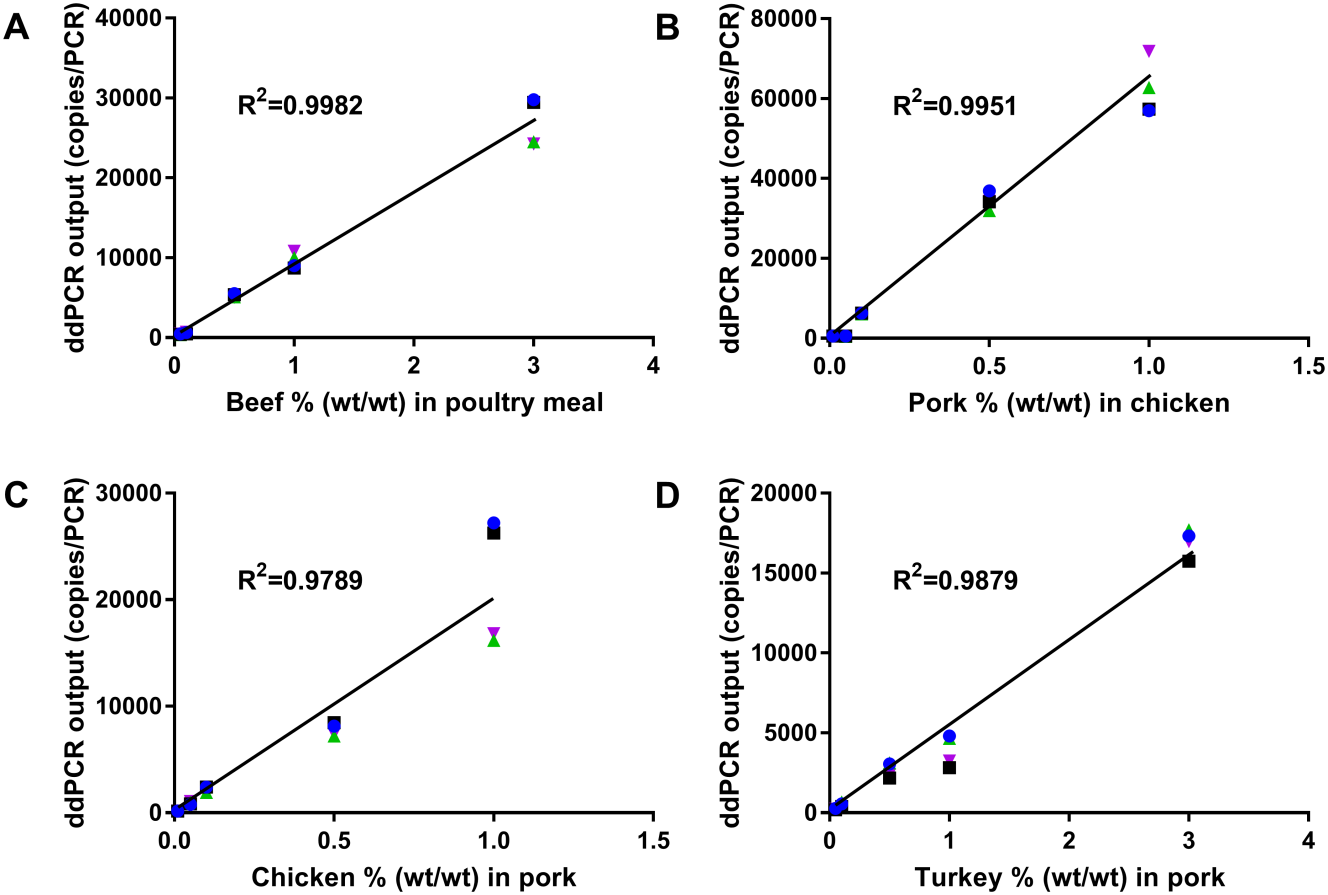
Linear regression between ddPCR output (copies/PCR) and target animal species concentration (% wt/wt) in fortified heat-processed samples. (A) beef in poultry meal, (B) pork in chicken, (C) chicken in pork, and (D) turkey in pork. Shown are results from four replicates in green, blue, purple and black.

Limit and range of quantification of the complete analytical processes were determined based on data from fortified heat-processed food/feed samples at different concentrations (0.005% to 10.00%, wt/wt) of beef in poultry meal, pork in chicken, chicken in pork or turkey in pork. The quantification range was found to be 0.05–3.00% (wt/wt) for heat-processed beef and turkey and 0.01–1.0% (wt/wt) for heat-processed pork and chicken with the coefficient of determination (R^2^) of 0.979–0.998 (p-value < 0.0001 for beef, pork and turkey, and p-value = 0.003 for chicken) (Fig 2). When the spiking levels were higher than 1.0 or 3.0% (wt/wt), the target molecule copies per droplet were above 1.5 (or 30000 copies/PCR), and the curves exhibited a plateau shape. This level was close to the theoretical upper limit of the ddPCR platform for quantification [42].

S2 Fig represents an example illustrating how the range of quantification of the bovine ddPCR assay was determined. For spiking levels above 3% the curve was no longer linear (S2A Fig) while it was linear for spiking levels up to 3% (S2B Fig). For spiking levels below 0.05%, the assay was not able to differentiate among 0%, 0.005% and 0.01% (S2C Fig).

### Repeatability and reproducibility

**Fig 3:**
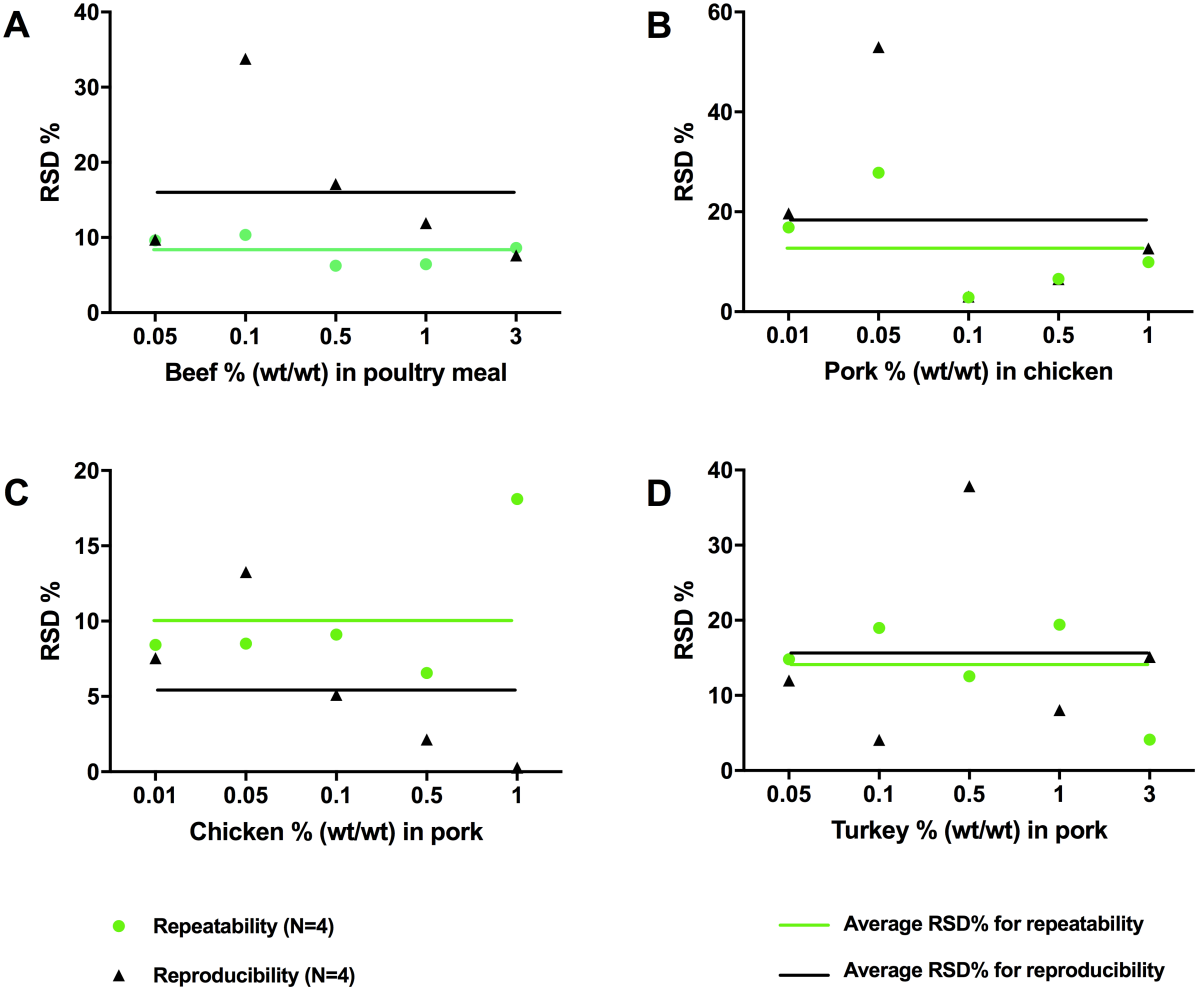
Repeatability and reproducibility of the ddPCR methods. Relative standard deviations (RSD%) are presented for fortified heat-processed (A) beef in poultry meal, (B) pork in chicken, (C) chicken in pork and (D) turkey in pork. Lines represent average RSD% for repeatability between DNA extractions and reproducibility between different operators. The data points cover all five spiking levels as labeled for each of the target species tested.

**Fig 4:**
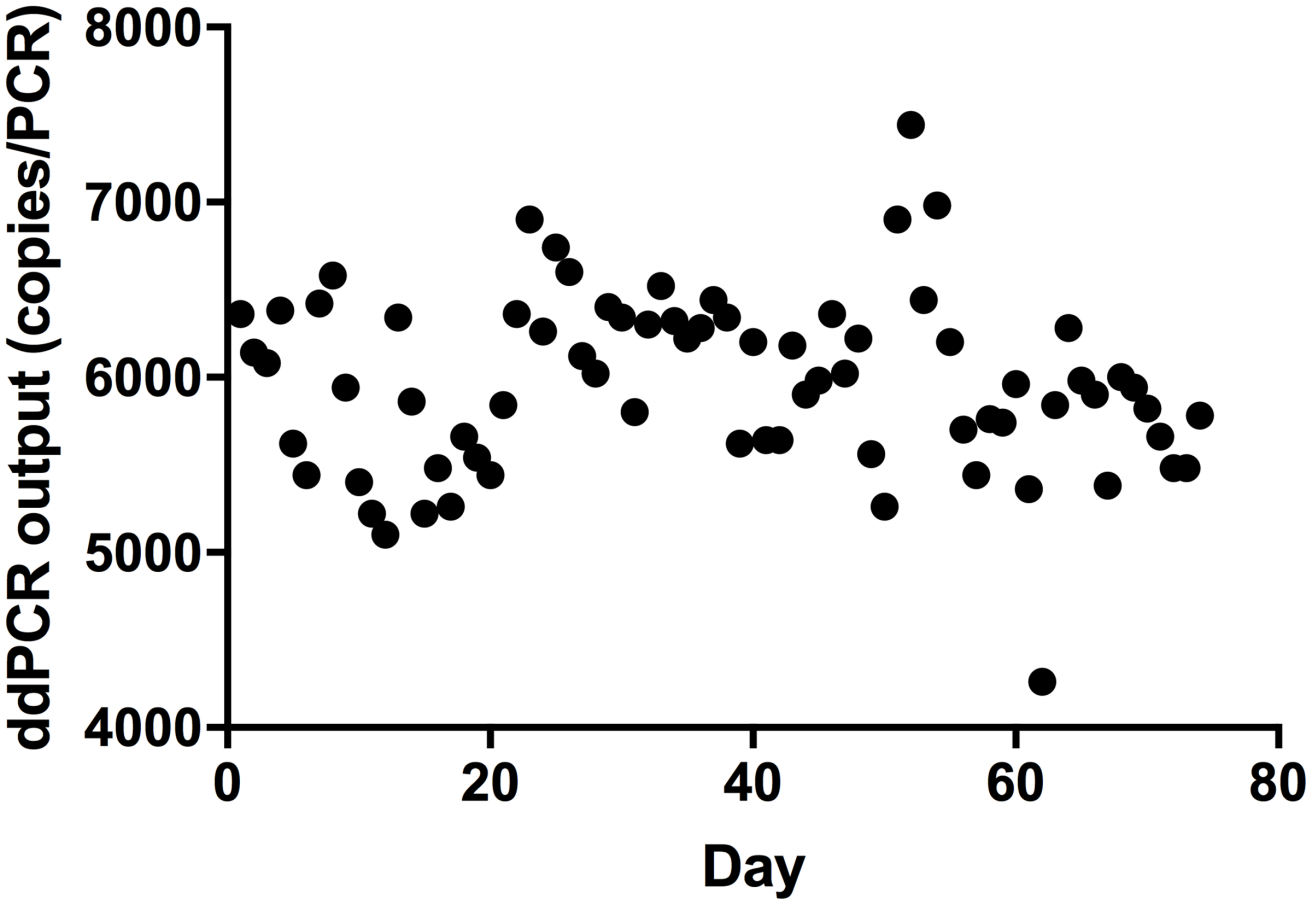
Internal validation of ddPCR for quantification of bovine DNA in a fortified poultry meal feed sample. The results were obtained from 74 consecutive runs conducted on different days. The variation (RSD %) was 8.6.

The ddPCR methods were tested for repeatability using replicated DNA extractions, PCRs and by using fortified heat-processed meat or feed samples. With few exceptions, the relative standard deviations (RSD) calculated for repeatability between PCR replicates and between DNA extractions were below 20% (Fig 3). The reproducibility of the methods was determined using three independent experiments conducted on different days and by different operators and was found to be below 20% of RSD with few exceptions (Fig 3). The higher RSD % observed in a few cases may have been caused mainly by variation in sample preparation. For example, the higher RSD at 0.1% for the bovine target (Fig 3) may have been caused by the heterogeneous nature of the feed matrix which contained small bone particles. The reproducibility of ddPCR was further monitored over time by making a daily control chart using the bovine ddPCR as an example. Fig 4 shows the ddPCR output of a fortified bovine positive control sample tested in 74 consecutive runs with each of the runs being performed on a different day. The variations (RSD = 8.6%) were within the reported technical range of 20% for the ddPCR platform (Fig 4).

### Matrix effect

**Fig 5:**
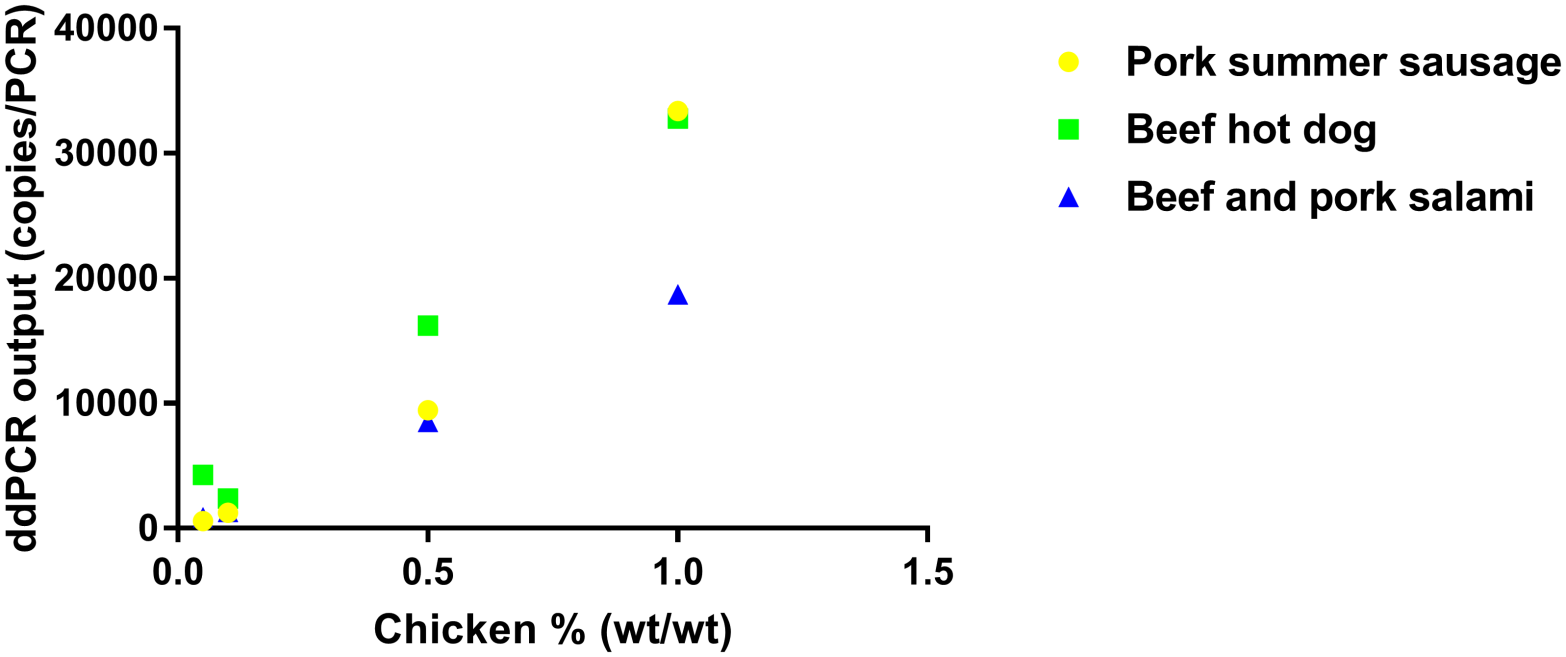
An example showing the effect of sample matrix on ddPCR output (copies/PCR). A cooked chicken sample was spiked in pork summer sausage, beef hot dog or beef and pork salami matrices.

Matrix effect was evaluated using a heat-processed animal tissue sample spiked into food or feed matrices. A matrix effect was observed in some of the fortified samples. For example, there was an over 45% difference in ddPCR output when spiking chicken into beef and pork salami as compared to chicken spiked into beef hot dogs (Fig 5).

### Robustness

The ddPCR assays were evaluated using DNA prepared from fortified samples of beef, pork and chicken within their quantification ranges under different DNA storage conditions, prolonged PCR reagent shelf life and using different PCR machines. RSD (%) ranges obtained under the different conditions were 2.0–22.6, 1.5–14.9 and 1.7–13.1 for the bovine, porcine and chicken ddPCR assays respectively (S2 Table). The RSD (%) values were within the acceptable range of quantitative accuracy, indicating that the assays can be performed using either fresh or frozen DNA, either fresh or old (close to the end of shelf life) reagent and using different PCR machines without compromising the results.

### Effect of DNA degradation on ddPCR readings

The effect of DNA degradation on ddPCR output was tested on both raw and cooked beef DNA after 3 different degradation treatments: Taq I restriction enzyme digestion, 2 min sonication, and 10 min sonication. The ddPCR output from degradation-treated DNA was compared to untreated DNA. Taq I digestion was found to significantly reduce ddPCR output for both raw and cooked beef DNA. Sonication for 10 min resulted in over 4.5 fold reduction in ddPCR output for raw beef DNA, and approximately 20% reduction for cooked beef DNA while sonication for 2 min showed little effect on ddPCR output for either raw or cooked meat DNA (Table 3). In addition, heat treatment of the beef tissue sample under the commonly used autoclave condition (15 min at 121°C) also resulted in over 3 fold reduction in the ddPCR output (Table 3).

**Table 3:** Effect of DNA degradation on ddPCR readings.

Sample type		Copies/PCR upon different treatments			
(0.032 ng DNA in AE Buffer)	Replicate	No treatment	Taq I digestion	2 min sonication	10 min sonication
Raw beef DNA	1	6800	4400	6000	1380
	2	6760	4360	6520	1480
Cooked beef DNA	1	2040	1360	1800	1520
	2	1820	1300	1680	1600

### Result calculation and interpretation

The ddPCR provides an absolute quantification of target DNA without relying on a standard curve. Specifically, the ddPCR output is in copies of input DNA. The correlation between the amount of DNA from fresh tissue and their copy numbers from the ddPCR was y = 291235x+103.18 for bovine, y = 188491x + 205.78 for porcine, y = 181118x−467.71 for chicken, and y = 398422x−240.49 for turkey (where y is the copy number and x is ng of DNA) (Fig 1). Based on these correlations, DNA mass of an amplified target from a sample can be calculated from its copy number. For example, the 3.0% (wt/wt) heat-treated beef in poultry meal feed resulted in 27000 copies which is equivalent to 0.0924 ng bovine DNA, or 0.23% in DNA mass/DNA mass. The quantitative range of 0.05%–3.00% (wt/wt) for the bovine target is equivalent to 0.0025–0.23% (DNA mass/DNA mass). When the % value is lower than the LOQ (0.0025% DNA mass or 0.05% dry weight of the sample), the result is reported to be <LOQ; when the % value is higher than 0.23% DNA mass or 3.00% dry weight, the DNA sample needs to be diluted to the quantitative range and retested.

The IC readings were used to normalize PCR outputs affected by variabilities in the PCR procedures. However, normalization of the ddPCR outputs to the IC readings did not affect the result if the IC readings were within 20% variation as compared to the expected values. S3 Fig illustrates an example of normalized and un-normalized results from the bovine ddPCR assay, which resulted in a p-value of 0.86 (n = 50) from paired t-test.

## Discussion

Food authentication continues to be of interest in an era of globalization as more reports and studies demonstrate a high incidence of adulteration and/or mislabeling. Here we describe and validate ddPCR- based assays for quantitative analysis of bovine, porcine, chicken and turkey DNA in food and feed. The ddPCR assays were evaluated systematically for their specificity, limit/range of quantification, repeatability and reproducibility, matrix effect and robustness. Other researchers have reported ddPCR assays for meat species quantification previously, including ddPCR assays for quantifying pork and chicken species [24], and for testing beef, pork and horse in meat products [4]. In this paper, we extended the detection scope to include beef, pork, chicken and turkey, investigated impact of sample nature and processing on results and enhanced the methods by integrating an internal control into the ddPCR assays to ensure data reliability.

The ddPCR assays described here were demonstrated to be specific upon testing 10–11 samples containing the target species and 45–46 samples belonging to 18 non-target species. The linear quantification range of these methods was 0.26–176 pg/PCR for fresh meat tissue DNA and 0.01–1.0% (wt/wt) for porcine and chicken ddPCR, and 0.05–3.0% (wt/wt) for bovine and turkey ddPCR for fortified heat-processed food and feed. Floren et al [4] also reported a ddPCR limit of quantification of 0.01% for mixed meat products. It is advised that the limits of quantification may not be comparable since meat samples were prepared by autoclaving for 15 minutes at 121°C in this study while cooking conditions to prepare the meat samples were not provided in the previous publication [4]. When the target species levels were above 1.0 or 3.0% (wt/wt), the linear regression curves exhibited a plateau. This result was expected as this level is considered close to the theoretical upper limit (30000 droplets) of the ddPCR platform for quantification. The dynamic quantification range for ddPCR is narrower than that of qPCR. However, dilution of the DNA preparations will allow the methods to quantify the target species above the upper limit of 1.0 or 3.0%. For example, representative samples containing 10.0% of bovine species were diluted and tested; ddPCR outputs proportional to the dilutions were observed (results not shown). Similar dilution practice was also reported by other researchers previously [24]. The repeatability and reproducibility of these methods were within 20% of RSD in general. Overall, the results indicate that the ddPCR assays can be used to reliably generate quantification results of the target DNA, especially for monitoring partial species substitution, and cross-contamination against pre-determined cut-off values.

The target sequences used for meat species detection in this study were mitochondrial DNA (mtDNA), which are widely used in animal species detection in complex food and feed samples [4]. The mitochondrial DNA genes were selected as the ddPCR targets in this study due to their presence in high copy numbers in animal species, achieving higher sensitivity and facilitating detection of trace quantities in food and feed matrices, especially after processing and severe DNA degradation [4, 43]. The effect of tissue type on ddPCR output was investigated in this study. We found that fresh bovine heart and pig liver resulted in 1.35 and 3 fold more target copies per unit weight of DNA in the bovine and porcine ddPCR respectively as compared to the fresh muscle samples. The effect of tissue type on ddPCR output is likely caused by the fact that the number of mitochondria per cell varies with tissue type [4, 43]. As reported previously, liver cells were found to contain approximately 3 times more mitochondria than muscle cells [44]. An alternative would be to use single copy genes (SCG) as amplification targets [43]. However, assays developed based on SCG may suffer from reduced sensitivity while high sensitivity is desired for applications where products are deeply processed and low tolerance is expected, such as testing for traces of bovine in feed or pork in Halal food, or monitoring product cross-contamination during production.

The ddPCR assays developed in this study contained an internal control (IC) to ensure reliability of the results, to normalize variabilities in the PCR procedures and to safeguard against false negatives due to factors such as PCR inhibition or reaction failure. The internal control plasmid DNA was added at a level to generate approximately 1700±20% copies per ddPCR reaction. At this IC concentration, no amplification competition was observed between the IC and the target DNA. Competitive amplification between the detection target and the IC was observed in ddPCR when more copies of IC were used and the target copy number was lower than that of IC in a reaction (S1 Table). The PCR test will pass the quality control only if the internal control results in the expected output with a ≤20% variation. Internal controls are used or required for qPCR assays [45] while the use of an internal control in ddPCR for meat species detection has not been a common practice in previous studies [4, 24]. The internal control created in this study can also be used in the format of recombinant *E*. *coli* cells. The IC cells can be added to food or feed samples, co-extracted and then co-amplified with the target, not only for monitoring inhibition or failure of amplification, but also for monitoring DNA extraction efficiency and calculating recovery.

Matrix effect was observed when testing fortified samples, or heat-processed animal tissue spiked into different food and feed sample matrices, such as sausages and hot dogs. However, the ddPCR output remained in linear correlation with target concentration. The matrix effect observed here may be explained by different components in the samples such as fats, presence of microbial population and also by the heterogeneous nature of the food and feed samples. Systematic matrix effect can be corrected by creating calibration curves or normalizing the data to the internal control when the IC is included in the sample before DNA extraction. Validation must be conducted in order to accurately quantify a target species in a matrix of different nature.

It is desirable to use “reference materials” in a validation study that are prepared under conditions close to production of the food or feed under testing. Meat materials were prepared in-house in this study due to lack of available reference materials representative of typical industrial meat and feed processing. Variations are expected from fortified materials prepared in different labs. Certified reference materials are needed to ascertain comparable results among different methods or different labs. These reference materials will help overcome limitations from using different food and feed materials in validation studies and extend the ability of ddPCR for use as a reliable quantitative technique, facilitating the establishment of consensus methods for food and feed testing. They will also ensure equivalency of results and support laboratory proficiency testing needs.

Food and feed production processes can cause severe DNA degradation due to heat or physical damage, resulting in several fold reduction in ddPCR outputs as compared to those from fresh tissues, as observed in this study. The effect of DNA degradation on ddPCR results was also observed with procedures that may be used in sample analysis, such as heating and sonication of a sample and restriction enzyme digestion of a DNA template. We found that heat treatment of a beef tissue sample by autoclaving for 15 min resulted in over 3 fold reduction in the ddPCR readings. Prolonged (e.g. 10 min) sonication of the template DNA resulted in underestimation (e.g. 4.5 fold reduction) of the target. Restriction enzyme digestion of genomic DNA templates has been recommended in the QX 200 experiment protocol for ddPCR in the manufacturer’s manual; however, Taq I digestion of the template in this study was found to significantly reduce ddPCR output although there was no Taq I restriction site within the amplicon. The prolonged incubation of the template during Taq I digestion (65°C for 1 hr) may have resulted in degradation within the amplicon. These findings emphasize the importance of minimising DNA degradation in analytical processes to ensure that the quantification numbers reflect the true nature of the samples. As shown, food processing can potentially result in underestimation of target species.

## Conclusions

The ddPCR assays described in this report met the accepted performance criteria of the ddPCR platform. The advantages of the methods include their high sensitivity, and ability to reliably quantify low concentration of DNA in a high background DNA without using standard curves. The internal control developed in this study can be used to monitor the PCR procedures and is recommended to be included in ddPCR assays to assess recovery and correct matrix effect. The methods can be used for quantitative analysis of bovine, porcine, chicken and turkey DNA in food and feed in validated matrices, particularly for products that are deeply processed or degraded and in which trace amount of foreign meat species is not tolerated. Standard reference material should be developed in collaboration with industry to mirror common production processes. The ddPCR methods can be implemented in routine testing to identify food fraud and to monitor the prohibited animal species in feed chain with enhanced sensitivity, accuracy and precision without reliance on standard curves.

## Supporting information

S1 Table Optimizing the concentration of internal control used in ddPCR assays. (PDF)

S2 Table Results of robustness study. (PDF)

S1 Fig Evaluating the repeatability of the ddPCR assay for the internal control (IC). IC was tested 16 times. The RSD% was 5.53.
(TIFF)

S2 Fig Example illustrating how the limit/range of quantification of the bovine ddPCR assay was determined. (A) ddPCR results for fortified beef in poultry meal at 0, 0.005, 0.01, 0.05, 0.1, 0.5, 1.0, 3.0, 5.0, and 10.0% (wt/wt). The curve exhibited a plateau when beef content was over 3.0%. (B-C) are subset data of (A) where (B) shows beef in poultry meal at 0, 0.005, 0.01, 0.05, 0.1, 0.5, 1.0, and 3.0% (wt/wt). After removing the 5.0 and 10.0% data points, the curve was linear. The upper limit was thus determined to be 3.0%. (C) shows beef in poultry meal at 0, 0.005, 0.01, 0.05, and 0.1% (wt/wt). The assay was unable to differentiate among 0, 0.005, and 0.01% of beef. The lower limit was thus determined to be 0.05%. The linear relationship was established between 0.05 and 3.0% beef as shown in Fig 2A. Each concentration was tested in 12 replicates.
(TIFF)

S3 Fig Evaluating the effect of normalizing the ddPCR output using the internal control (IC). ddPCR results were obtained from testing fortified heat-processed beef in poultry meal without normalization to IC (A) and after normalization to IC (B).
(TIFF)

## References

[1] Spink J, Moyer DC. Defining the public health threat of food fraud. J Food Sci. 2011;76(9):R157–R63. doi: 10.1111/j.1750-3841.2011.02417.x 22416717 10.1111/j.1750-3841.2011.02417.x

[2] Chuah L-O, He XB, Effarizah ME, Syahariza ZA, Shamila-Syuhada AK, Rusul G. Mislabelling of beef and poultry products sold in Malaysia. Food Control. 2016;62:157–64.

[3] Zacharisen MC. Severe allergy to chicken meat. Wis Med J. 2006;105(5):50–2. 16933414

[4] Floren C, Wiedemann I, Brenig B, Schutz E, Beck J. Species identification and quantification in meat and meat products using droplet digital PCR (ddPCR). Food Chem. 2015;173:1054–8. doi: 10.1016/j.foodchem.2014.10.138 25466124 10.1016/j.foodchem.2014.10.138

[5] O'Mahony PJ. Finding horse meat in beef products—a global problem. QJM. 2013;106(6):595–7. doi: 10.1093/qjmed/hct087 23625529 10.1093/qjmed/hct087

[6] Di Pinto A, Bottaro M, Bonerba E, Bozzo G, Ceci E, Marchetti P, et al Occurrence of mislabeling in meat products using DNA-based assay. J Food Sci Technol. 2015;52(4):2479–84. doi: 10.1007/s13197-014-1552-y 25829637 10.1007/s13197-014-1552-y PMC4375227

[7] Bottaro M, Marchetti P, Mottola A, Shehu F, Di Pinto A. Detection of mislabeling in packaged chicken sausages by PCR. Albanian J Agric Sci. 2014:455.

[8] Tartaglia M, Saulle E, Pestalozza S, Morelli L, Antonucci G, Battaglia PA. Detection of bovine mitochondrial DNA in ruminant feeds: a molecular approach to test for the presence of bovine-derived materials. J Food Prot. 1998;61(5):513–8. 9709219 10.4315/0362-028x-61.5.513

[9] Kumar A, Kumar RR, Sharma BD, Gokulakrishnan P, Mendiratta SK, Sharma D. Identification of species origin of meat and meat products on the DNA basis: a review. Crit Rev Food Sci Nutr. 2015;55(10):1340–51. doi: 10.1080/10408398.2012.693978 24915324 10.1080/10408398.2012.693978

[10] Sentandreu MÁ, Sentandreu E. Authenticity of meat products: Tools against fraud. Food Res Int. 2014;60:19–29.

[11] Ali ME, Kashif M, Uddin K, Hashim U, Mustafa S, Che Man YB. Species authentication methods in foods and feeds: the present, past, and future of halal forensics. Food Anal Method. 2012;5(5):935–55.

[12] Ansfield M, Reaney S, Jackman R. Production of a sensitive immunoassay for detection of ruminant and porcine proteins, heated to >> 130°C at 2.7 bar, in compound animal feedstuffs. Food Agric Immunol. 2000;12(4):273–84.

[13] Haider N, Nabulsi I, Al-Safadi B. Identification of meat species by PCR-RFLP of the mitochondrial *COI* gene. Meat Sci. 2012;90(2):490–3. doi: 10.1016/j.meatsci.2011.09.013 21996288 10.1016/j.meatsci.2011.09.013

[14] Ali ME, Razzak MA, Hamid SBA. Multiplex PCR in species authentication: probability and prospects—A review. Food Anal Method. 2014;7(10):1933–49.

[15] Mafra I, Ferreira IMPLVO, Oliveira MBPP. Food authentication by PCR-based methods. Eur Food Res Technol. 2008;227(3):649–65.

[16] Calvo JH, Rodellar C, Zaragoza P, Osta R. Beef-and bovine-derived material identification in processed and unprocessed food and feed by PCR amplification. J Agric Food Chem. 2002;50(19):5262–4. 12207458 10.1021/jf020051a

[17] Ekins J, Peters SM, Jones YL, Swaim H, Ha T, La Neve F, et al Development of a multiplex real-time PCR assay for the detection of ruminant DNA. J Food Prot. 2012;75(6):1107–12. doi: 10.4315/0362-028X.JFP-11-415 22691479 10.4315/0362-028X.JFP-11-415

[18] Frezza D, Favaro M, Vaccari G, Von-Holst C, Giambra V, Anklam E, et al A competitive polymerase chain reaction–based approach for the identification and semiquantification of mitochondrial DNA in differently heat-treated bovine meat and bone meal. J Food Prot. 2003;66(1):103–9. 12540188 10.4315/0362-028x-66.1.103

[19] Krčmář P, Renčová E. Identification of bovine-specific DNA in feedstuffs. J Food Prot. 2001;64(1):117–9. 11198432 10.4315/0362-028x-64.1.117

[20] Prado M, Berben G, Fumière O, van Duijn G, Mensinga-Kruize J, Reaney S, et al Detection of ruminant meat and bone meals in animal feed by real-time polymerase chain reaction: result of an interlaboratory study. J Agric Food Chem. 2007;55(18):7495–501. doi: 10.1021/jf0707583 17725317 10.1021/jf0707583

[21] Rodríguez MA, García T, González I, Asensio L, Hernández PE, Martín R. PCR identification of beef, sheep, goat, and pork in raw and heat-treated meat mixtures. J Food Prot. 2004;67(1):172–7. 14717369 10.4315/0362-028x-67.1.172

[22] Zhang C-L, Fowler MR, Scott NW, Lawson G, Slater A. A TaqMan real-time PCR system for the identification and quantification of bovine DNA in meats, milks and cheeses. Food Control. 2007;18(9):1149–58.

[23] Huggett JF, Foy CA, Benes V, Emslie K, Garson JA, Haynes R, et al Guidelines for minimum information for publication of quantitative digital PCR experiments. Clin Chem. 2013;59(6):892–902. 23570709 10.1373/clinchem.2013.206375

[24] Cai YC, Li X, Lv R, Yang JL, Li J, He YP, et al Quantitative analysis of pork and chicken products by droplet digital PCR. BioMed Res Int. 2014;2014(1):1–6. 10.1155/2014/810209 PMC4163444 25243184

[25] Baker M. Digital PCR hits its stride. Nat Meth. 2012;9(6):541–4.

[26] Hindson BJ, Ness KD, Masquelier DA, Belgrader P, Heredia NJ, Makarewicz AJ, et al High-throughput droplet digital PCR system for absolute quantitation of DNA copy number. Anal Chem. 2011;83(22):8604–10. doi: 10.1021/ac202028g 22035192 10.1021/ac202028g PMC3216358

[27] Dingle TC, Sedlak RH, Cook L, Jerome KR. Tolerance of droplet-digital PCR vs real-time quantitative PCR to inhibitory substances. Clin Chem. 2013;59(11):1670–2. doi: 10.1373/clinchem.2013.211045 24003063 10.1373/clinchem.2013.211045 PMC4247175

[28] Nadauld L, Regan JF, Miotke L, Pai RK, Longacre TA, Kwok SS, et al Quantitative and sensitive detection of cancer genome amplifications from formalin fixed paraffin embedded tumors with droplet digital PCR. Translational medicine (Sunnyvale, Calif). 2012;2(2):1–12. 10.4172/2161-1025.1000107 PMC3653435 23682346

[29] Henrich TJ, Gallien S, Li JZ, Pereyra F, Kuritzkes DR. Low-level detection and quantitation of cellular HIV-1 DNA and 2-LTR circles using droplet digital PCR. J Virol Methods. 2012;186(1):68–72. 22974526 10.1016/j.jviromet.2012.08.019 PMC3517891

[30] Kelley K, Cosman A, Belgrader P, Chapman B, Sullivan DC. Detection of methicillin-resistant *Staphylococcus aureus* by a duplex droplet digital PCR assay. J Clin Microbiol. 2013;51(7):2033–9. doi: 10.1128/JCM.00196-13 23596244 10.1128/JCM.00196-13 PMC3697713

[31] Rothrock MJ, Hiett KL, Kiepper BH, Ingram K, Hinton A. Quantification of zoonotic bacterial pathogens within commercial poultry processing water samples using droplet digital PCR. Advances in Microbiology. 2013;3(05):403.

[32] Strain MC, Lada SM, Luong T, Rought SE, Gianella S, Terry VH, et al Highly precise measurement of HIV DNA by droplet digital PCR. PloS one. 2013;8(4):e55943 doi: 10.1371/journal.pone.0055943 23573183 10.1371/journal.pone.0055943 PMC3616050

[33] Morisset D, Štebih D, Milavec M, Gruden K, Žel J. Quantitative analysis of food and feed samples with droplet digital PCR. PloS one. 2013;8(5):e62583 doi: 10.1371/journal.pone.0062583 23658750 10.1371/journal.pone.0062583 PMC3642186

[34] ANSI. Species-Level Identification of Animal Cells through Mitochondrial Cytochrome c Oxidase Subunit 1 (CO1) DNA Barcodes 2015 [cited 2017]. ANSI/ATCC ASN-0003-2015:[http://webstore.ansi.org/RecordDetail.aspx?sku=ANSI%2FATCC+ASN-0003-2015.

[35] Udomsil N, Chen S, Rodtong S, Yongsawatdigul J. Quantification of viable bacterial starter cultures of *Virgibacillus sp*. and *Tetragenococcus halophilus* in fish sauce fermentation by real-time quantitative PCR. Food Microbiol. 2016;57:54–62. doi: 10.1016/j.fm.2016.01.004 27052702 10.1016/j.fm.2016.01.004

[36] Tanabe S, Hase M, Yano T, Sato M, Fujimura T, Akiyama H. A real-time quantitative PCR detection method for pork, chicken, beef, mutton, and horseflesh in foods. Biosci Biotechnol Biochem. 2007;71(12):3131–5. doi: 10.1271/bbb.70683 18071237 10.1271/bbb.70683

[37] Krcmar P, Rencova E. Identification of species-specific DNA in feedstuffs. J Agric Food Chem. 2003;51(26):7655–8. doi: 10.1021/jf034167y 14664524 10.1021/jf034167y

[38] Abuzinadah OH, Yacoub HA, El Ashmaoui HM, Ramadan HA. Molecular detection of adulteration in chicken products based on mitochondrial 12S rRNA gene. Mitochondrial DNA. 2015;26(3):337–40. doi: 10.3109/19401736.2013.840593 24102598 10.3109/19401736.2013.840593

[39] AOAC983.18. Moisture in meat. AOAC Method 2008;950.46B (1991 revision).

[40] AOAC. Meat and meat products-Preparation of test sample procedures. 2006.

[41] AOAC. AOAC Method 1983. 983.18.

[42] Pinheiro LB, Coleman VA, Hindson CM, Herrmann J, Hindson BJ, Bhat S, et al Evaluation of a droplet digital polymerase chain reaction format for DNA copy number quantification. Anal Chem. 2012;84(2):1003–11. doi: 10.1021/ac202578x 22122760 10.1021/ac202578x PMC3260738

[43] Ballin NZ, Vogensen FK, Karlsson AH. Species determination—Can we detect and quantify meat adulteration? Meat Sci. 2009;83(2):165–74. doi: 10.1016/j.meatsci.2009.06.003 20416768 10.1016/j.meatsci.2009.06.003

[44] Rodríguez MA, García T, González I, Asensio L, Mayoral B, López-Calleja I, et al Identification of goose, mule duck, chicken, turkey, and swine in foie gras by species-specific polymerase chain reaction. J Agric Food Chem. 2003;51(6):1524–9. doi: 10.1021/jf025784+ 12617577 10.1021/jf025784+

[45] Diez-Valcarce M, Cook N, Hernández M, Rodríguez-Lázaro D. Analytical application of a sample process control in detection of foodborne viruses. Food Anal Method. 2011;4(4):614–8.

