# Supplementary material for: Droplet digital polymerase chain reaction (ddPCR) assays integrated with an internal control for quantification of bovine, porcine, chicken and turkey species in food and feed
Source: PLoS One. 2017 Aug 10;12(8):e0182872. doi: 10.1371/journal.pone.0182872 (PMC5552122; doi:10.1371/journal.pone.0182872)
Supplement: S1 Table — (PDF) [file pone.0182872.s001.pdf]

S1 Table. Optimizing the internal control (IC) concentration in ddPCR assays

IC concentration (fg/µl) + Bovine DNA (2.0 fg/µL) in a 1:4 ratio	Target copies/PCR	IC copies/PCR
60000	1.6	194000
60000	3.4	198000
6000	100	46600
6000	128	47120
600	322	4600
600	308	4540
60	362	450
60	444	444
6	442	48
6	420	58
0.60	392	6.4
0.60	324	4.6
0.06	378	0
0.06	394	1.6
