# Supplementary material for: Droplet digital polymerase chain reaction (ddPCR) assays integrated with an internal control for quantification of bovine, porcine, chicken and turkey species in food and feed
Source: PLoS One. 2017 Aug 10;12(8):e0182872. doi: 10.1371/journal.pone.0182872 (PMC5552122; doi:10.1371/journal.pone.0182872)
Supplement: S2 Table — (PDF) [file pone.0182872.s002.pdf]

S2 Table. Results of robustness study. RSD (%) of ddPCR assays obtained from testing fortified cooked meat samples under different conditions in terms of DNA storage (fresh vs frozen at -20ºC for 3 weeks), PCR reagent shelf life (fresh vs close to expiry date) and PCR machine age (new vs old)

	RSD% of different ddPCR assays		
**Detection target**	**Bovine**	**Porcine**	**Chicken**
**Range tested (% wt/wt)**	0.05-3.0	0.01-1.0	0.01-1.0
**DNA storage**	6.7-22.6	4.7-12.0	1.7-11.5
**PCR reagent shelf life**	5.6-13.3	3.1-9.5	1.7-11.5
**PCR machine age**	2.0-13.3	1.5-14.9[a]	2.9-13.1

[a]There was one outlier with RSD of 59% at 0.05%. It is expected that it was due to heterogeneous nature of the sample.
