# Supplementary figures and images for: Droplet digital polymerase chain reaction (ddPCR) assays integrated with an internal control for quantification of bovine, porcine, chicken and turkey species in food and feed
Source: PLoS One. 2017 Aug 10;12(8):e0182872. doi: 10.1371/journal.pone.0182872 (PMC5552122; doi:10.1371/journal.pone.0182872)

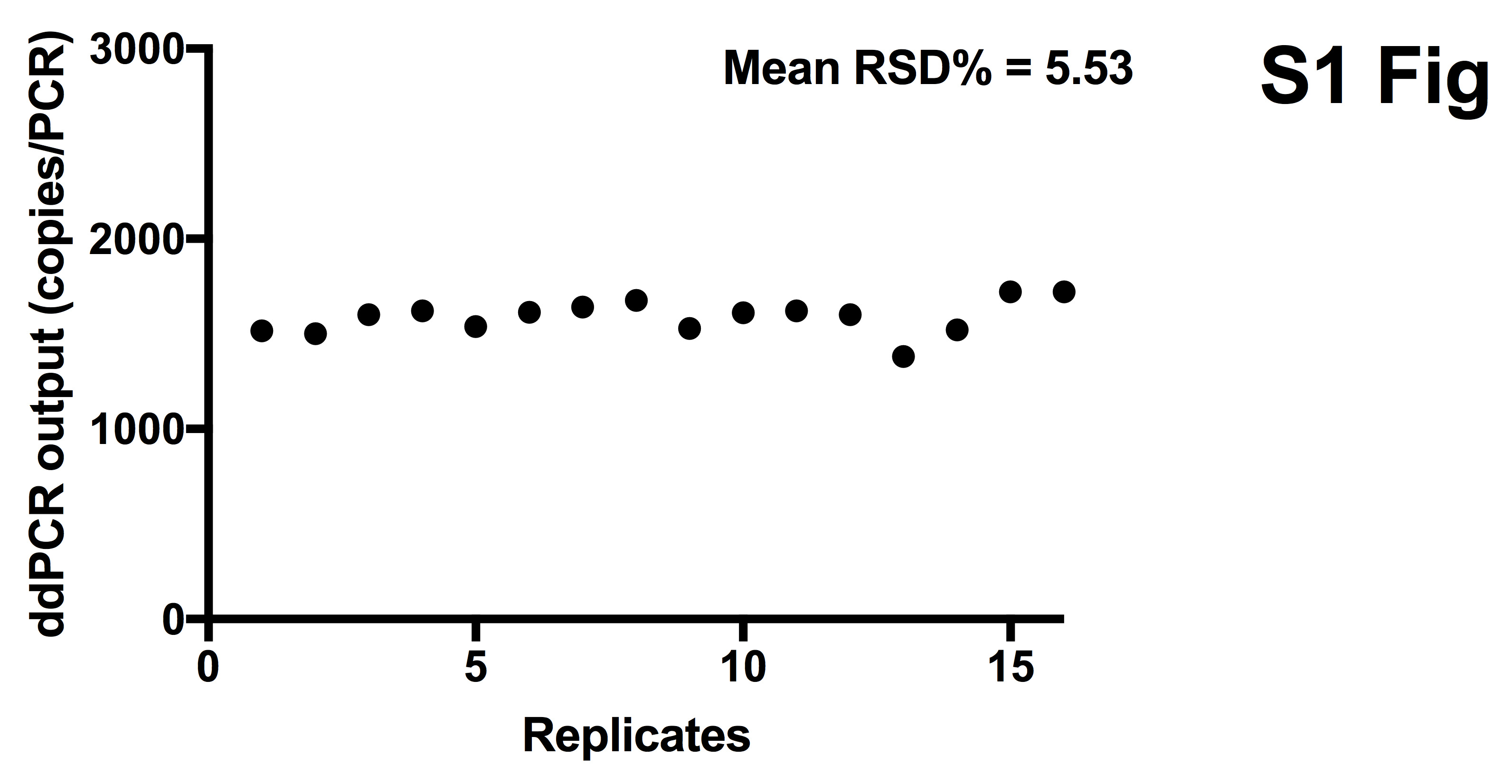

Supplement: S1 Fig — IC was tested 16 times. The RSD% was 5.53. (TIFF) [file pone.0182872.s003.tiff]

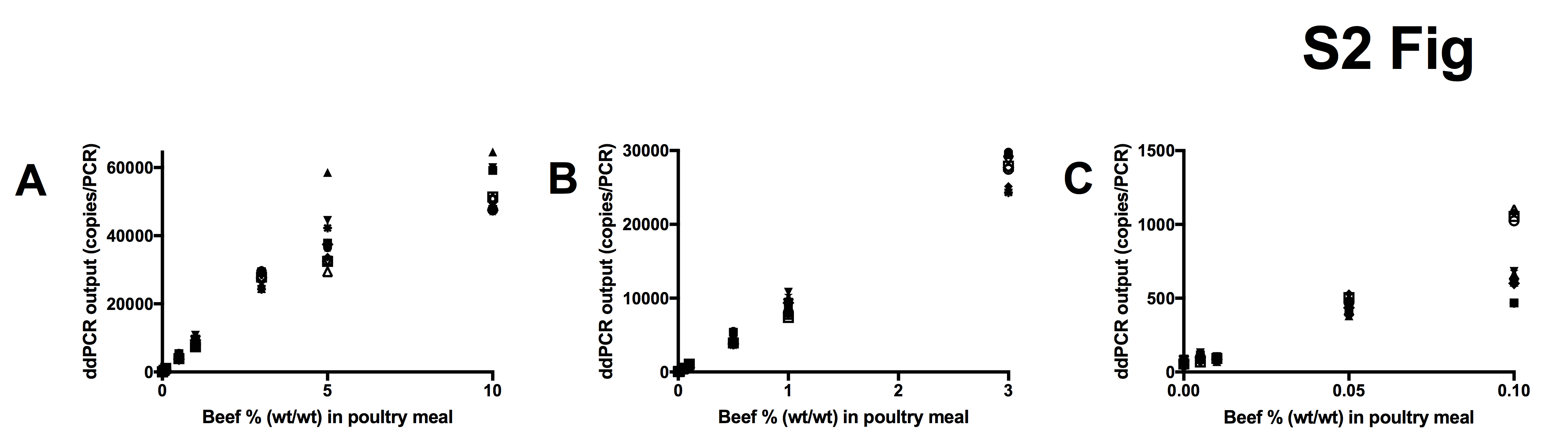

Supplement: S2 Fig — (A) ddPCR results for fortified beef in poultry meal at 0, 0.005, 0.01, 0.05, 0.1, 0.5, 1.0, 3.0, 5.0, and 10.0% (wt/wt). The curve exhibited a plateau when beef content was over 3.0%. (B-C) are subset data of (A) where (B) shows beef in poultry meal at 0, 0.005, 0.01, 0.05, 0.1, 0.5, 1.0, and 3.0% (wt/wt). After removing the 5.0 and 10.0% data points, the curve was linear. The upper limit was thus determined to be 3.0%. (C) shows beef in poultry meal at 0, 0.005, 0.01, 0.05, and 0.1% (wt/wt). The assay was unable to differentiate among 0, 0.005, and 0.01% of beef. The lower limit was thus determined to be 0.05%. The linear relationship was established between 0.05 and 3.0% beef as shown in Fig 2A. Each concentration was tested in 12 replicates. (TIFF) [file pone.0182872.s004.tiff]

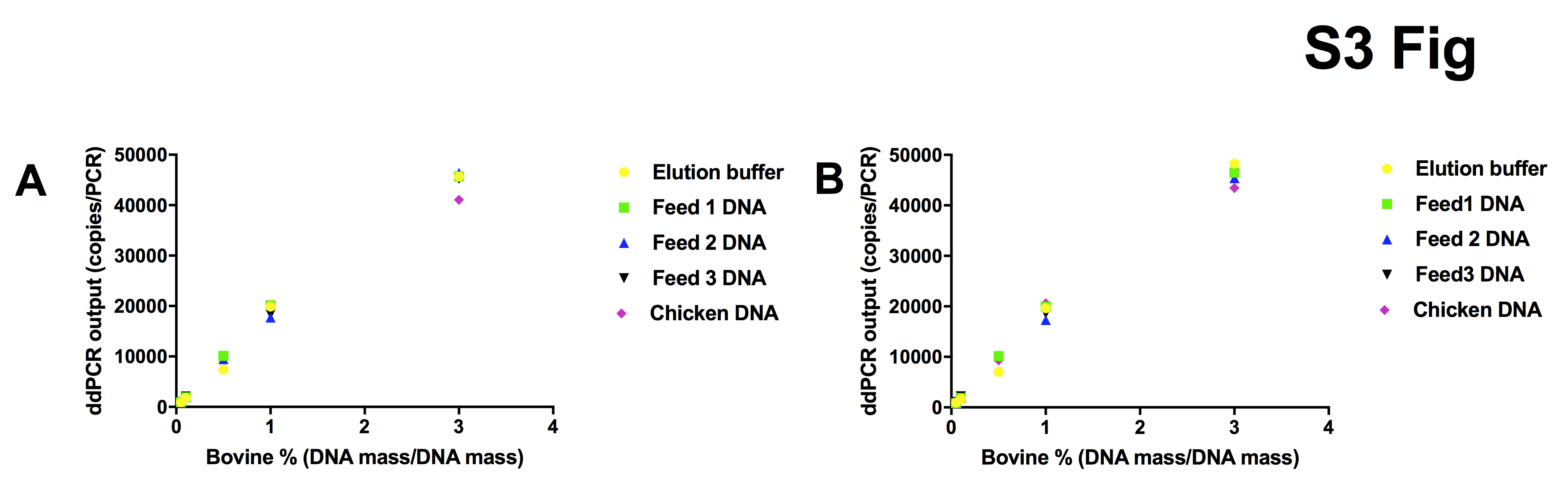

Supplement: S3 Fig — ddPCR results were obtained from testing fortified heat-processed beef in poultry meal without normalization to IC (A) and after normalization to IC (B). (TIFF) [file pone.0182872.s005.tiff]
